# Comparative assessment of CBCT and digital radiography in quantifying periapical pathology (in vitro study)

**DOI:** 10.1186/s12903-026-09816-z

**Published:** 2026-09-25

**Authors:** Abdelmonem Dahrous, Hassan Mohamed Abouelkheir, Esraa A. Elnadoury

**Affiliations:** 1 Bachelor of Dentistry, FacultyofDentistry, Alexandria University, Alexandria, Egypt; 2 Department of Oral Medicine, Oral Periodontology, Oral Diagnosis and Oral Radiology, Faculty of Dentistry, Alexandria University, Alexandria, Egypt; 3 Department of Oral Medicine, Oral Periodontology, Oral Diagnosis and Oral Radiology Faculty of Dentistry, Alexandria University, Alexandria, Egypt

**Keywords:** Digital Periapical X-Ray, CBCT, Periapical Lesion, Length, PSP

## Abstract

**Objective:**

To compare the diagnostic accuracy of phosphor plate-based digital periapical radiography (DPR) and CBCT in quantifying periapical lesions compared to the reference standard.

**Materials and methods:**

This in vitro study included 45 premolar teeth with simulated periapical lesions created in dry human mandibles. The lesions were categorized into three groups according to size. All specimens were imaged using DPR with the paralleling technique (70–80kVp, 8mA, 0.16–0.32s). CBCT imaging was performed using the i-CAT Next Generation system (120kVp, 5mA, 26.9s, 0.25mm voxel size). Lesion lengths obtained from both imaging modalities were compared with the actual lesion dimensions (reference standard) to assess diagnostic accuracy. Intra-examiner reliability was evaluated.

**Results:**

Group I's mean lesion length was 1.2mm, Group II's was 1.5mm, and Group III's was 1.8mm. In comparison to the reference standard, PR frequently overestimated the length of lesions. CBCT, on the other hand, showed better accuracy, and greater consistency. A propensity to underestimate minor lesions was indicated by the mean CBCT measurement of 1.07 ± 0.20mm at 1.2 mm group (*p* = 0.020). At 1.5mm, CBCT assessed 1.72 ± 0.19mm (*p* = 0.001) while no significant difference observed at 1.8mm (*p* = 0.065).

**Conclusions:**

While CBCT is more reliable than PR, its precision is influenced by lesion size, performing most accurately with larger periapical pathologies.

**Clinical relevance:**

Periapical radiography should be interpreted with caution when assessing periapical pathology, as it may lead to overestimation of lesion size. In contrast, CBCT can be regarded as the reference standard, especially for lesions larger than 1.5mm.

## Introduction

In endodontics, radiography is a crucial diagnostic tool for both disease diagnosis and assessment of healing. Pre- and postoperative radiographs are typically compared to determine the treatment outcome in addition to clinical findings [1].

Periapical Radiography (PR) is considered the clinically preferred method for the assessment, diagnosis, and management of endodontic disease [2, 3]. However, its limitations are well-established, primarily due to the two-dimensional nature of the images, anatomical noise, and geometric distortion. Research has shown that the detection of periapical lesions within cancellous bone using PR can be challenging, as the dense overlying cortical bone can obscure underlying pathological changes [4].

Several in vitro and phantom-based studies have reported that photostimulable phosphor (PSP) systems demonstrate image quality and technical performance comparable to, or slightly superior to, CCD/CMOS sensors, particularly in terms of sharpness, contrast resolution, angulation, and receptor placement [5].

Cone Beam Computed Tomography (CBCT) three-dimensional nature mitigates some of the disadvantages of DPR. But there are drawbacks to this approach, including greater radiation doses (19–368 mSv) compared to DPR (2–9 mSv), high levels of noise and scattering [6–8].

When comparing DPR with CBCT for lesion detection, conventional periapical imaging failed to identify small periapical lesions, whereas CBCT demonstrates superior diagnostic accuracy, particularly for lesions exceeding 0.8–1 mm in size [9, 10]. However, evidence from a recent clinical trial suggests that the diagnostic accuracy of both modalities remains questionable under certain conditions [11].

Nevertheless, it was stated that due to the lack of a reference standard, there is currently insufficient high-quality clinical evidence to support the diagnostic effectiveness of CBCT compared with digital periapical [11]. This implies the need for in vitro studies that directly compare radiographic modalities with the actual (gold standard). Moreover, current research frequently prioritizes the detection of periapical lesions; however, the linear and volumetric quantification of these defects remains under-explored, despite its paramount importance in monitoring healing and determining surgical complexity [10, 12, 13].

The present study aimed to compare DPR using photostimulable phosphor (PSP) receptors with CBCT in quantifying periapical lesions of varying sizes. This study's null hypothesis postulates that CBCT and digital DPR demonstrate equivalent diagnostic accuracy in the assessment of periapical lesion length.

## Materials and methods

This in-vitro study was designed and reported in accordance with the CRIS (Checklist for Reporting In-Vitro Studies) 2014 guidelines [14]. Ethical approval was obtained from the Ethics Committee of the Faculty of Dentistry, Alexandria University (IRB no 00010556 IORG 0008839, Ethics committee No: 1048–03/2025).

Sample size estimation was performed assuming a confidence level of 95% and a study power of 80% based on the findings reported by Sheikhi et al. [12]. The required sample size was calculated using Rosner’s method [15] via G*Power 3.1.9.7 software (Heinrich-Heine-Universität Düsseldorf, Germany) [16]. Based on the mean difference between dependent measurements and a maximum standard deviation of 1.5, an effect size of 0.867 was obtained. A minimum of 13 samples per group was therefore required; this was increased to 15 samples per group to compensate for potential processing losses, yielding a total sample of 45 specimens (15 × 3 groups).

A total of forty-five freshly extracted sound human mandibular premolars, extracted for orthodontic purposes, were obtained from the Outpatient Clinics of the Faculty of Dentistry, Alexandria University.

Teeth were allocated into three groups according to simulated lesion size: Group I ≈1.0 mm; Group II ≈1.5 mm; and Group III ≈2.0 mm.

**Fig. 1:**
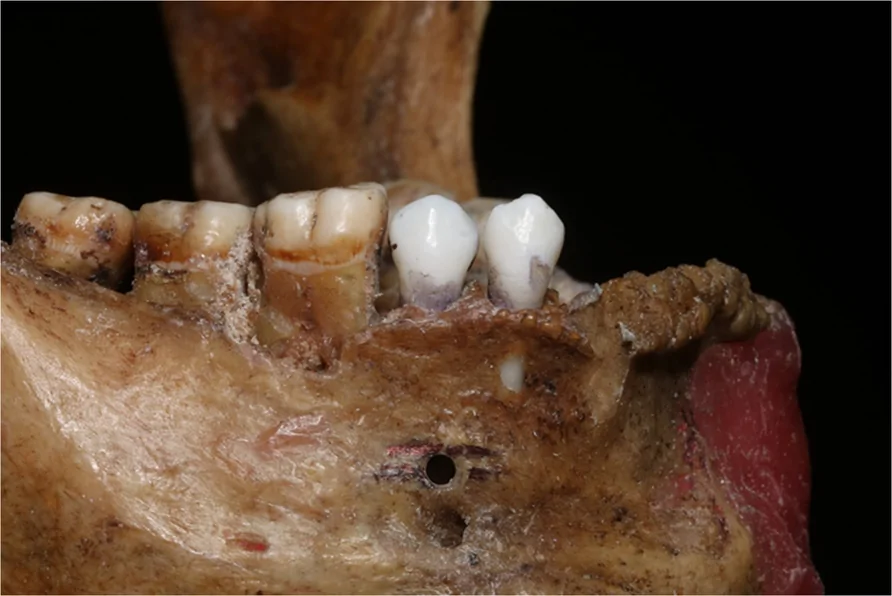
Periapical lesion after lesion creation and positioning of the tooth within the socket

Teeth were mounted in a dry human mandible [17] to simulate anatomical structures. Standardized periapical defects were created at the end of the socket parallel to its long axis (Fig. 1) using round tungsten carbide burs (Komet Brasseler, Lemgo, Germany) mounted in a contra-angle handpiece operated at 4,000–12,000 rpm. Following creation of each experimental defect, its physical vertical dimension was measured directly using a calibrated digital caliper (Insize Basic Digital Caliper, Insize Co., China). This direct physical measurement was predefined as the reference standard against which the periapical radiography and CBCT measurements were compared. To simulate soft tissue attenuation, a 2–3 mm layer of pink modeling wax (Cavex Set Up Wax, Cavex Holland BV, Haarlem, Netherlands) was applied around the mandible.

**Fig. 2:**
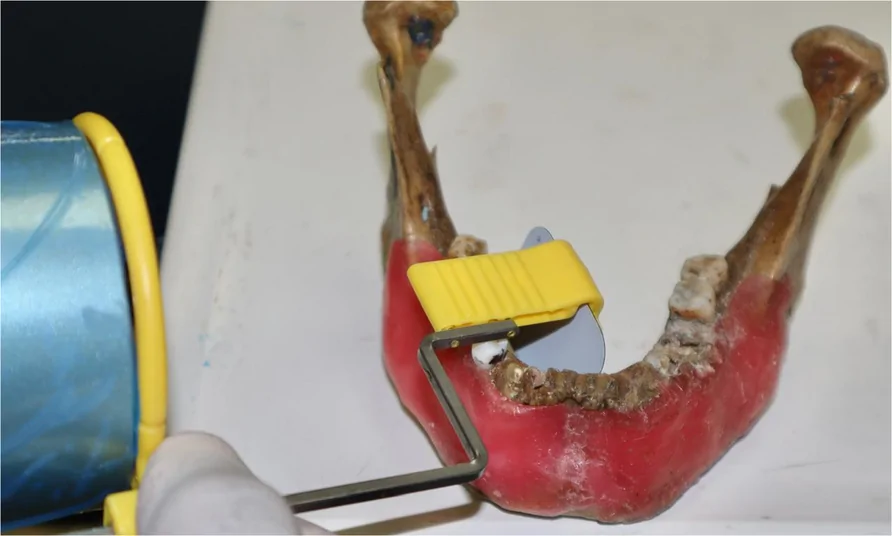
Placement of the PSP plate during acquisition of a periapical radiograph using XCP

**Fig. 3:**
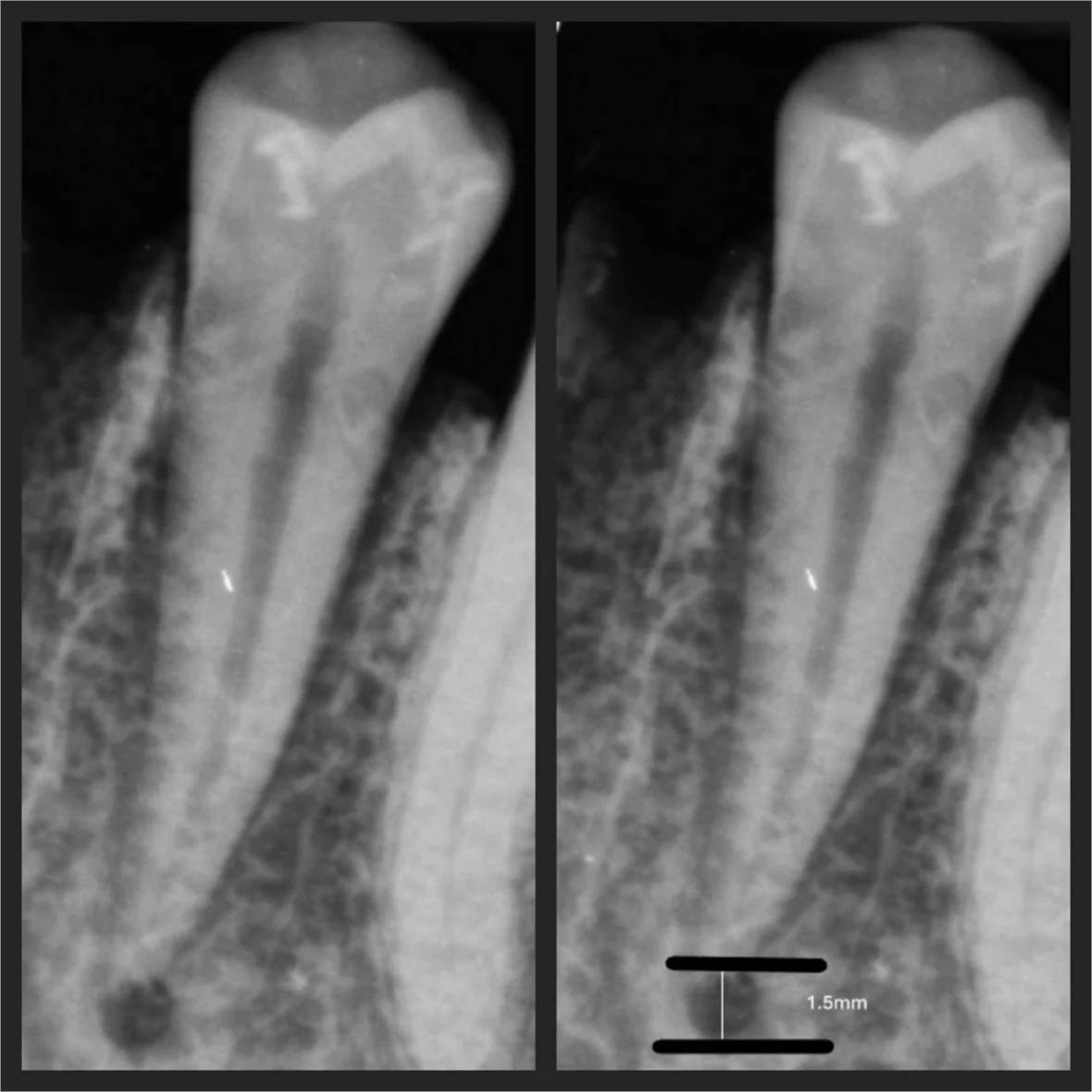
Standardized digital periapical x-ray measurement

Digital periapical radiographs were acquired using a phosphor storage plate (PSP) system (CS 7200, Carestream Dental LLC, Atlanta, GA, USA). Imaging was performed using a standardized parallel technique using an XCP film holder (Rinn® XCP, Dentsply Sirona, York, PA, USA) to ensure reproducibility (Fig. 2). Exposures were performed using a dental X-ray unit (Dentsply Sirona Inc., Charlotte, NC, USA) with the following exposure parameters: 70–80 kVp, 8 mA, 0.16–0.32 s. Images were scanned and viewed using Carestream Dental Imaging Software (Carestream Dental LLC). Brightness and contrast adjustments were allowed for optimal visualization. Imaging orders were randomized and coded. The observer was blinded regarding the lesion size group. Superior and inferior radiopaque lesion margins were identified, and vertical lesion height was measured using the software digital ruler tool (Fig. 3).

**Fig. 4:**
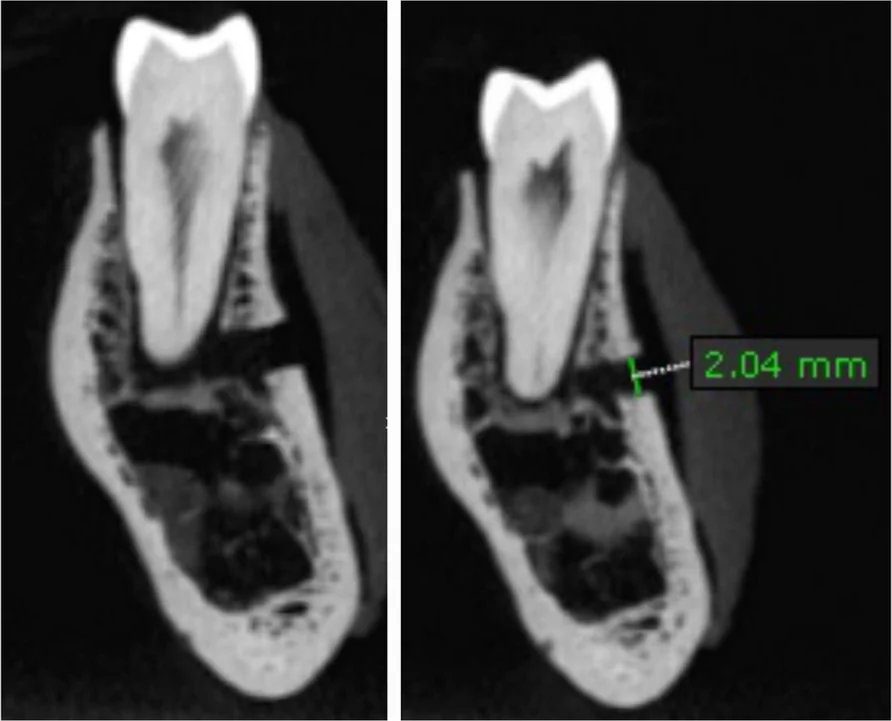
CBCT measurements in cross-sectional cut

CBCT scans were acquired using an I-CAT Next Generation unit (Imaging Sciences International, Hatfield, PA, USA) with standardized exposure parameters (120 kVp, 5 mA, 26.9 s, 0.25 mm voxel size). Images were reconstructed and viewed using OnDemand3D™ software (Cybermed Inc., Seoul, South Korea). Dental Volume Reconstruction (DVR) mode was used for measurement. A panoramic curve was drawn along the arch at the cemento-enamel junction level to generate cross-sectional slices. Reference lines were positioned parallel to the long axis of the tooth. Vertical lesion dimensions were measured (Fig. 4).

Measurements were performed by a trained radiologist. Prior to experimental image evaluation, calibration was conducted using four specimens. The observer independently evaluated these images, and the results were reviewed and verified by a senior oral and maxillofacial radiologist (E.A) to establish standard diagnostic criteria. Intra-observer consistency was further confirmed by re-evaluating 10% of the study specimens after a two-week washout period. The intra-examiner reproducibility was assessed for CBCT and periapical X-ray measurements.

### Statistical analysis

Data were analyzed using IBM SPSS Statistics version 25 (IBM Corp., Armonk, NY, USA) and MedCalc version 20.218 (MedCalc Software Ltd., Ostend, Belgium). Data validation was performed prior to analysis. Complementary statistical analyses were performed using Stata version 11 (StataCorp LP, College Station, TX, USA).

Continuous measurements were summarized using the mean and standard deviation. The distributions of the paired measurement differences were assessed using the Shapiro–Wilk test and inspection of Q–Q plots. Normally distributed paired differences were analyzed using the paired-samples t test, whereas the Wilcoxon signed-rank test was used when the normality assumption was not satisfied [18, 19].

For each lesion-size group, measurements obtained using digital periapical radiography and CBCT were compared with the corresponding physical lesion dimension measured using the digital caliper as the reference standard. The two imaging modalities were also compared directly using paired analyses.

Because all specimens contained experimentally created periapical lesions and the study outcome was the continuous measurement of lesion length rather than the binary detection or classification of lesions, sensitivity, specificity, positive predictive value, negative predictive value, receiver operating characteristic curve analysis, and area under the curve were not applicable. Measurement performance was therefore evaluated by quantifying measurement error, mean bias, 95% confidence intervals, and limits of agreement relative to the physical reference measurements [20].

To account for multiple inferential comparisons, p values were adjusted using the Holm procedure [21]. Measurement agreement was evaluated using Bland–Altman analysis. The mean difference, representing measurement bias, and its 95% confidence interval were calculated together with the 95% limits of agreement, defined as the mean difference ± 1.96 standard deviations of the paired differences [22]. Two-sided statistical tests were used, and the predefined level of statistical significance was set at *p* < 0.05 [23].

## Results

The mean lesion length in Group I was 1.2 mm, in Group II 1.5 mm, and in Group III 1.8 mm. The Intraclass Correlation Coefficient (ICC) was 0.983 (95% CI: 0.932–0.996; *p* < 0.001) for periapical radiography and 0.981 (95% CI: 0.924–0.995; *p* < 0.001) for CBCT, indicating excellent reliability.

Periapical radiography consistently overestimated lesion size relative to the reference standard. By contrast, CBCT demonstrated measurements closer to the reference standard. At 1.2 mm, the mean CBCT measurement was 1.07 ± 0.20 mm, which was significantly lower than the reference value (*p* = 0.020), indicating a tendency to underestimate small lesions. At 1.5 mm, CBCT measured 1.72 ± 0.19 mm, which was slightly but significantly greater than the reference size (*p* = 0.001) while no significant difference was observed at 1.8 mm (*p* = 0.065), indicating improved accuracy with larger lesions (Tables 1 and 2).

**Table 1:** Comparison of lesions size using different radiographic measurement techniques with the reference standard

**Radiographic techniques**	**Lesion size (reference standard)**					
	**1.2 mm**		**1.5 mm**		**1.8 mm**	
	**Mean ± SD**	*P*** value**	**Mean ± SD**	***P* value**	**Mean ± SD**	***P *value**
Periapical	1.54 ± 0.09	< 0.001*	1.75 ± 0.14	< 0.001*	2.48 ± 0.13	< 0.001*
CBCT	1.07 ± 0.20	0.020*	1.72 ± 0.19	0.001*	1.94 ± 0.27	0.065
Mean difference	0.47 ± 0.20	-	0.04 ± 0.23	-	0.54 ± 0.33	-
*p* value#	< 0.001*	-	0.560	-	< 0.001*	-

^*^Statistically significant difference at *p* value < 0.05, #Paired t test

**Table 2:** Agreement Between Periapical Radiography, CBCT, and reference Standard Using Bland–Altman Analysis

**Radiographic techniques**	**Lesion size**	**Mean difference (95% CI)**	***p* value (H**_**0**_**: Mean = 0)**	**Agreement limits**	
				**Lower limit (95% CI)**	**Upper limit (95% CI)**
Reference	1.2 mm	−0.34 (−0.39, −0.29)	< 0.001*	−0.52	−0.16
Standard vs				(−0.61, −0.43)	(−0.25, −0.07)
Periapical	1.5 mm	−0.25 (−0.33, −0.18)	< 0.001*	−0.52	0.01
				(−0.65, −0.39)	(−0.12, 0.14)
	1.8 mm	−0.68 (−0.75, −0.61)	< 0.001*	−0.94	−0.42
				(-1.07, −0.81)	(−0.55, −0.29)
Reference	1.2 mm	0.13 (0.02, 0.24)	0.019*	−0.25	0.52
Standard vs				(−0.44, −0.06)	(0.33, 0.71)
CBCT	1.5 mm	−0.22 (−0.32, −0.11)	0.001*	−0.59	0.16
				(−0.77, −0.41)	(−0.03, 0.34)
	1.8 mm	−0.14 (−0.29, 0.01)	0.065	−0.67	0.39
				(−0.94, −0.41)	(0.13, 0.66)
Periapical vs	1.2 mm	0.47 (0.36, 0.58)	< 0.001*	0.08	0.87
CBCT				(−0.11, 0.28)	(0.67, 1.06)
	1.5 mm	0.04 (−0.9, 0.17)	0.560	−0.42	0.49
				(−0.65, −0.20)	(0.27, 0.72)
	1.8 mm	0.54 (0.26, 0.72)	< 0.001*	−0.10	1.18
				(−0.41, 0.22)	(0.86, 1.49)

^*^Statistically significant difference at *p* value < 0.05, CI: Confidence Interval

Bland–Altman analysis demonstrated systematic differences between the radiographic techniques. Compared with the reference standard, periapical radiography consistently overestimated lesion size, with progressively larger negative biases at greater lesion dimensions (–0.34 mm at 1.2 mm to –0.68 mm at 1.8 mm). CBCT showed smaller mean differences with the reference standard; it slightly underestimated small lesions (bias = + 0.13 mm at 1.2 mm), showed a modest overestimation at 1.5 mm (–0.22 mm), and was not significantly different at 1.8 mm (–0.14 mm, *p* = 0.065), indicating better agreement at larger lesion size. When comparing periapical radiography with CBCT, periapical values were consistently higher, with significant positive biases at 1.2 mm and 1.8 mm, but no significant difference at 1.5 mm.

Notably, CBCT exhibited narrower limits of agreement, reflecting greater measurement consistency and closer concordance with the reference standard. While for periapical X-ray, the data points are relatively close to the limits of agreement, indicating less consistency than CBCT, with differences remaining negative across the measurement range, showing no clear trend toward smaller or larger means, but consistently overestimating**.** Although a statistically acceptable agreement was observed between periapical radiography and CBCT, periapical measurements showed reduced precision and a consistent overestimation relative to CBCT.

## Discussion

Accurate evaluation of periapical lesion size is clinically relevant, as lesion dimensions have been proposed as prognostic indicators for periapical disease progression and treatment outcome [24]. The findings of the present study demonstrated that CBCT provided higher accuracy in the quantitative assessment of periapical pathology; therefore, the null hypothesis was rejected.

Conventional periapical radiography remains the gold-standard imaging modality for the diagnosis and follow-up of apical periodontitis [25]. However, it presents inherent limitations. To minimize geometric errors in the current study, the paralleling technique was employed for all periapical radiographs, which is known to improve image reproducibility and dimensional accuracy [26].

The selected size ranges were based on prior radiographic studies demonstrating that periapical lesions below 1 mm are radiographically challenging to detect on two-dimensional radiographs, while CBCT detects lesions as small as 0.5–1 mm due to the lack of superimposition [27, 28]. Lesions above 1.5 mm become radiographically detectable, but measurement accuracy varies with defect size due to magnification and noise effects [27, 29]. Further studies indicated that CBCT struggles with very tiny (< 0.8–2 mm) lesions [27, 30]. Therefore, three clinically relevant size categories were selected to simulate early periapical defects [31, 32].

Dental modeling wax applied in this study is widely accepted in ex vivo radiographic models as a tissue-equivalent material due to its similar radiation attenuation and scatter properties relative to oral soft tissues, thereby providing image contrast conditions that closely mimic clinical settings [33].

In the present study, Periapical radiography demonstrated mild overestimation in the first two groups and moderate overestimation in the 1.8 mm group. In contrast, CBCT showed mild underestimation in Group I and mild overestimation in Group II, with no significant difference in the third group. Similar overestimation patterns were reported when using large field-of-view CBCT in the study by Sheikhi et al. [12], where lesions measuring approximately 1.7 mm exhibited significantly higher measurement variability compared to larger lesions. These findings suggest that CBCT demonstrates higher accuracy for lesions exceeding 1.8 mm in length. This may be attributed to the difficulty in identifying and controlling measurement points in very small lesions. As in CBCT, identifying the "starting point" of a lesion is subjective. Studies show that even experienced radiologists have low-to-moderate agreement when manually measuring the linear extent of cancellous-confined lesions [34–36].

The diagnostic superiority and accuracy of CBCT observed in our study align closely with findings reported by Tsai et al. [27] in which CBCT showed higher diagnostic accuracy than periapical radiography, with AUCs of 0.767 for Kodak CBCT and 0.753 for Morita CBCT versus 0.584 for PA radiography. While Karkle et al. [37] reported that CBCT measured lesions approximately 0.61 mm larger than periapical radiograph (PA) measurements on average, our findings demonstrated the opposite pattern, with CBCT yielding consistently lower measurement values than PA.

DPR overestimation may be related to its lower spatial resolution. Importantly, the observed overestimation contradicts the traditional assumption that PR predominantly underestimates lesion extent due to cortical masking [12]. Instead, the current data indicate that once lesions become radiographically visible, their apparent boundaries may be exaggerated, potentially leading to misinterpretation of disease severity.

Across the literature, overestimation is common for periapical radiographs in implant, tooth-length, and some bone-level measurements, but other applications show underestimation or mixed error rather than a uniform bias [38–42]. Cassetta et al. [43] reported that periapical radiographs significantly overestimated peri-implant marginal bone levels compared to direct surgical measurements, with a mean radiographic-surgical difference of 0.45 mm. These findings closely mirror our periapical results.

Standardized pre- and post-treatment periapical radiographs remain clinically valuable for assessing changes over time [44], as the relative dimensional differences can still reflect true biological progression or healing, particularly when CBCT is not indicated due to cost, radiation exposure, or accessibility considerations.

Current literature supports the use of CBCT as a targeted adjunct in diagnostically challenging or inconclusive cases, rather than as a universally established first-line imaging modality [45]. However, the findings of the present study demonstrate superior diagnostic performance of CBCT in the assessment of periapical lesion length, suggesting its potential to serve as a reference imaging modality in the evaluation of apical periodontitis under controlled conditions.

Nevertheless, this recommendation should be interpreted with caution in clinical practice, taking into consideration radiation dose and adherence to the ALADAIP (As Low As Diagnostically Acceptable, being Indication-oriented and Patient-specific) principle [46]. Optimization strategies, including the use of a limited field of view and appropriate exposure parameters, are essential to minimize patient radiation exposure while maintaining diagnostic image quality.

The present study has several limitations that should be considered when interpreting the findings. First, it’s in vitro design limits direct extrapolation to clinical conditions, where factors such as patient movement, and soft tissue variability, may influence image quality and diagnostic performance. Second, the use of single-rooted premolars does not fully replicate the anatomical complexity of multirooted teeth, such as molars, which may affect the generalizability of the results. Additionally, the method used to create experimental lesions represents a limitation. Lesions were mechanically prepared using burs, resulting in standardized cavities with well-defined borders and regular contours. In contrast, naturally occurring periapical lesions typically present with diffuse margins and irregular shapes. This discrepancy may have facilitated lesion detection and measurement on imaging, potentially leading to an overestimation of diagnostic accuracy compared with true clinical scenarios. Furthermore, imaging was performed using Cone Beam Computed Tomography with a medium field of view. Future studies utilizing smaller fields of view are recommended to enhance spatial resolution and reduce radiation dose, in accordance with contemporary radiation protection principles. Finally, a limitation of the present study is that image evaluation was conducted by a single observer, preventing the assessment of inter-observer variability. Consequently, observer-specific bias cannot be entirely ruled out, and future studies involving multiple independent raters are recommended to confirm these findings.

## Conclusions

Within the limitations of this in vitro study, Cone Beam Computed Tomography demonstrated superior accuracy and reliability compared with digital periapical radiography in the assessment of periapical lesion length. Periapical radiography exhibited a tendency to overestimate lesion dimensions, with the magnitude of error increasing as lesion size increased. In contrast, CBCT measurements showed minimal deviation and a closer agreement with the reference standard.

These findings highlight the enhanced capability of CBCT for precise three-dimensional evaluation of periapical pathology. Accordingly, CBCT may be considered a reference imaging modality for the assessment of periapical lesion dimensions, particularly in situations where accurate measurement is critical for diagnosis and treatment planning and with lesions larger than 1.5 mm, while still adhering to radiation protection principles.

## Data Availability

The datasets created or analyzed during the current study are not publicly available but are available from the corresponding author upon reasonable request.
